# Supplementary material for: Metabolomic Signature Discriminates Normal Human Cornea from Keratoconus—A Pilot GC/MS Study
Source: Molecules. 2020 Jun 25;25(12):2933. doi: 10.3390/molecules25122933 (PMC7356237; doi:10.3390/molecules25122933)
Supplement: Supplementary file 1 [file molecules-25-02933-s001.pdf]

Supplementary material

Metabolomic Signature Discriminates Normal Human Cornea from Keratoconus—A Pilot GC/MS Study

Anna Wojakowska [1], Monika Pietrowska [2], Piotr Widlak [2], Dariusz Dobrowolski [3,4], Edward Wylęgała [4,5] and Dorota Tarnawska [5,6,*]

1 European Centre for Bioinformatics and Genomics, Institute of Bioorganic Chemistry Polish Academy of Sciences, Noskowskiego12/14, 61-704 Poznan, Poland; astasz@ibch.poznan.pl
2 Maria Sklodowska-Curie National Research Institute of Oncology, Gliwice Branch, Wybrzeze Armii Krajowej 15, 44-102 Gliwice, Poland; monika.pietrowska@io.gliwice.pl (M.P.); piotr.widlak@io.gliwice.pl (P.W.)
3 Department of Ophthalmology & Tissue and Cells Bank, St. Barbara Hospital, Trauma Center, Plac Medyków 1, 41-200 Sosnowiec, Poland; dardobmd@wp.pl
4 Chair and Clinical Department of Ophthalmology, Division of Medical Science in Zabrze, Medical University of Silesia, Panewnicka 65, 40-760 Katowice, Poland; wylegala@gmail.com
5 Department of Ophthalmology, District Railway Hospital, Panewnicka 65, 40-760 Katowice, Poland
6 Faculty of Science and Technology, Silesian Center for Education and Interdisciplinary Research, University of Silesia, 75 Pułku Piechoty 1A, 41-500 Chorzów, Poland
* Correspondence: dorota.tarnawska@us.edu.pl

Received: 25 May 2020; Accepted: 22 June 2020; Published: date

Table S1. Metabolites identified and quantified by GC/MS in keratoconus (KC) and healthy cornea (HC) samples.

No	Metabolite name	Class	Mol. mass	Rt	KC		HC	
					mean abundance	S.D.	mean abundance	S.D.
1	Lactic acid	α-hydroxy acids	88	06.19	1.70E−04	1.73E−04	1.38E−04	1.64E−04
2	Glycolic acid	α-hydroxy acids	88	06.34	2.65E−05	4.10E−05	6.18E−05	5.39E−05
3	Oxalic acid	Carboxylic acids	189	07.20	2.56E−04	3.039E−04	2.53E−04	2.01E−04
4	Urea	Others	189	08.56	1.22E−03	9.00E−04	1.48E−03	4.71E−04
5	Phosphoric acid	Others	299	09.21	6.77E−01	9.24E−02	5.69E−01	3.75E−02
6	Glycerol	Sugar alcohols	218	09.23	6.94E−04	4.70E−04	7.06E−04	4.99E−04
7	3-Indoleacetic acid	Others	130	09.39	3.21E−03	1.15E−03	3.41E−03	1.30E−03
8	Glycine	Amino acids	174	09.51	3.48E−03	2.14E−03	5.33E−03	7.32E−03
9	Succinic acid	Carboxylic acids	147	09.58	5.36E−03	1.21E−03	7.71E−03	1.41E−03

10	Serine	Amino acids	204	10.34	1.95E−03	2.38E−03	1.22E−03	3.95E−04
11	Benzoic acid	Carboxylic acids	215	10.37	1.19E−03	4.38E−04	1.80E−03	4.05E−04
12	Lactic acid dimer	α-hydroxy acids	88	10.54	7.54E−05	7.56E−05	1.64E−04	8.12E−05
13	L-Aspartic acid	Amino acids	232	12.38	3.45E−04	4.07E−04	1.90E−04	5.52E−05
14	L-5-Oxoproline	Amino acids	156	12.39	6.29E−03	9.94E−03	2.61E−03	8.44E−04
16	Octanoic acid 2-ethylhexyl ester	Fatty acid esters	84	14.53	3.62E−04	2.25E−04	4.63E−04	9.15E−05
17	Azelaic acid	Carboxylic acids	317	15.51	9.17E−05	3.76E−05	1.38E−04	6.83E−05
18	Citric acid	Carboxylic acids	273	16.03	2.51E−04	1.83E−04	4.47E−04	5.07E−04
19	Myristic acid	Fatty acids	285	16.25	1.12E−03	3.94E−04	1.75E−03	3.06E−04
20	D-Fructose	Saccharides	217	16.36	1.41E−03	1.12E−03	1.34E−03	4.90E−04
21	D-Psicose	Saccharides	307	16.42	3.10E−04	1.92E−04	2.85E−04	9.51E−05
22	D-Glucose	Saccharides	319	16.52	2.10E−03	2.18E−03	2.88E−03	7.89E−04
23	Decanoic acid. 2-ethylhexyl ester	Carboxylic acid esters	84	16.59	5.39E−04	2.58E−04	7.25E−04	1.85E−04
24	Palmitic acid methyl ester	Fatty acid esters	87	17.14	2.32E−03	1.04E−03	3.04E−03	6.91E−04
25	Sorbitol	Sugar alcohols	319	17.16	6.15E−04	7.34E−04	5.68E−05	4.93E−05
26	Pentadecanoic acid	Fatty acids	117	17.26	7.43E−04	2.62E−04	1.22E−03	3.31E−04
27	Hexadecanol	Alcohols	299	17.34	7.63E−04	1.88E−04	1.09E−03	1.80E−04
28	Talose	Saccharides	204	17.40	4.83E−04	4.25E−04	8.26E−04	5.60E−04
29	Gluconic acid	Carboxylic acids	435	17.54	5.62E−06	7.30E−06	N.D.	N.D.
30	Palmitoleic acid	Fatty acids	311	18.11	2.93E−04	1.45E−04	3.81E−04	2.08E−04
31	Palmitic acid	Fatty acids	313	18.24	4.25E−02	1.57E−02	7.24E−02	1.26E−02
32	Myo-Inositol	Sugar alcohols	305	18.47	9.41E−05	5.87E−05	1.49E−04	6.52E−05
33	Stearic acid. methyl ester	Fatty acid esters	87	19.11	1.16E−03	5.16E−04	1.50E−03	3.68E−04
34	Linoleic acid	Fatty acids	337	19.55	3.88E−04	3.79E−04	1.03E−03	4.99E−04
35	trans-9-Octadecenoic acid	Fatty acids	84	19.59	1.35E−03	7.10E−04	9.30E−04	1.26E−03
36	Petroselinic acid	Fatty acids	339	19.59	N.D.	N.D.	2.56E−03	3.24E−03
37	trans-13-Octadecenoic acid	Fatty acids	357	20.00	8.33E−04	1.71E−04	1.49E−03	2.11E−04
38	Stearic acid	Fatty acids	341	20.13	4.81E−02	1.77E−02	7.44E−02	1.68E−02
39	3-Chloropropionic acid. heptadecyl ester	Others	84	20.42	2.97E−04	1.39E−04	6.56E−04	1.31E−04
40	Myo-Inositol phosphate	Others	318	21.37	4.49E−03	4.41E−03	1.92E−03	5.32E−04
41	Arachidic acid	Fatty acids	369	21.53	4.78E−04	1.96E−04	6.28E−04	1.35E−04
42	Sucrose	Saccharides	361	23.22	2.34E−03	8.76E−04	3.63E−03	1.27E−03
43	Cholesterol chloroformate	Sterol esters	368	24.31	8.70E−05	8.29E−05	4.96E−04	2.35E−04
44	Cholesta-3.5-diene – isomer 1	Sterols	368	25.11	1.76E−04	1.63E−04	8.36E−04	4.03E−04
45	Cholesta-3.5-diene – isomer 2	Sterols	368	25.27	8.55E−04	7.59E−04	4.21E−03	2.01E−03

46	Cholesterol propionate	Sterol esters	351	25.27	1.20E−06	1.85E−06	N.D.	N.D.
47	Cholesterol	Sterols	133	27.34	1.96E−02	1.51E−02	5.68E−02	1.52E−02

N.D. – not detected, Rt – retention time (min), S.D. – standard deviation.
